# PIM3 Kinase: A Promising Novel Target in Solid Cancers

**DOI:** 10.3390/cancers16030535

**Published:** 2024-01-26

**Authors:** Pinar Atalay, Bulent Ozpolat

**Affiliations:** 1 Department of Nanomedicine, Houston Methodist Research Institute, Houston, TX 77030, USA; patalaydundar@houstonmethodist.org; 2 Methodist Neil Cancer Center, Houston, TX 77030, USA

**Keywords:** PIM3, PIM kinase inhibitors, targeted therapies, serine/threonine kinase, solid cancer, triple-negative breast cancer

## Abstract

**Simple Summary:**

*PIM3* is a serine/threonine kinase linked to various oncogenic processes and often overexpressed in solid cancers such as pancreatic, liver, colon, stomach, and breast cancers. Upregulation of PIM3 is associated with poor patient prognosis, and its inhibition leads to reduced cell proliferation, invasion, and in vivo tumor growth; thus, PIM3 represents an emerging novel therapeutic target in cancer. Although pan-PIM inhibitors have entered clinical trials in hematological cancers, they were not potent or specific enough or exhibited side effects; thus, currently, there is no FDA-approved inhibitor for targeting all PIMs or PIM3. The development of selective and effective PIM3 inhibitors may have a significant clinical impact and a novel potential therapeutic strategy for PIM3-driven solid and hematological cancers.

**Abstract:**

PIM3 (provirus-integrating Moloney site 3) is a serine/threonine kinase and belongs to the PIM family (PIM1, PIM2, and PIM3). PIM3 is a proto-oncogene that is frequently overexpressed in cancers originating from endoderm-derived tissues, such as the liver, pancreas, colon, stomach, prostate, and breast cancer. PIM3 plays a critical role in activating multiple oncogenic signaling pathways promoting cancer cell proliferation, survival, invasion, tumor growth, metastasis, and progression, as well as chemo- and radiation therapy resistance and immunosuppressive microenvironment. Genetic inhibition of PIM3 expression suppresses in vitro cell proliferation and in vivo tumor growth and metastasis in mice with solid cancers, indicating that PIM3 is a potential therapeutic target. Although several pan-PIM inhibitors entered phase I clinical trials in hematological cancers, there are currently no FDA-approved inhibitors for the treatment of patients. This review provides an overview of recent developments and insights into the role of PIM3 in various cancers and its potential as a novel molecular target for cancer therapy. We also discuss the current status of PIM-targeted therapies in clinical trials.

## 1. Introduction

PIM3 is a serine/threonine kinase that belongs to the provirus-integrating site Moloney murine leukemia virus (PIM) family, consisting of PIM1, PIM2, and PIM3 [1]. PIM proteins are crucial in various malignancies, including hematologic cancers and some solid tumors [2,3]. PIM1 overexpression is linked to poor clinical outcomes in patients with leukemia [4] and triple-negative breast cancer. Similarly, *PIM2* is overexpressed in both leukemia and solid tumors and stimulates the transcription of genes involved in cell survival, proliferation, and cell cycle progression [3]. *PIM3* was originally known as a kinase induced by depolarization (KID-1) in rat pheochromocytoma cells [5], but it was later renamed *PIM3* because of its sequence similarity with other PIM family proteins [6]. PIM3 belongs to the Ca^2+^/calmodulin-dependent protein kinase (CaMK) family and functions as a non-receptor serine/threonine kinase, similar to other PIM family members [7].

*PIM3* expression has been detected in multiple human tissues, such as the brain, kidney, heart, spleen, placenta, lung, skeletal muscle, peripheral blood leukocytes [8], and endothelial cells [9]. *PIM3* is highly expressed in tumor tissues, particularly in those derived from the endoderm, such as the liver, pancreas, colon, and stomach. *PIM3* overexpression has also been observed in triple-negative breast cancers and hematological malignancies. Higher *PIM3* expression is often correlated with shorter overall survival in patients with colorectal, pancreatic, and prostate cancer, hepatoblastoma, and breast cancer (Table 1) [5,10,11]. Silencing PIM3 inhibits the proliferation of various cancer cell lines in vitro and promotes apoptosis [8,12,13]. PIM3 has been shown to promote growth and angiogenesis of human pancreatic cancer cells in vivo in an orthotopic nude mouse model [14], and the inhibition of PIM3 kinase halts the growth of human tumors that were injected into nude mice, suggesting that PIM3 is a potential molecular target for cancer therapy [6]. This review highlights the structure and role of PIM3 as an oncogenic kinase in solid cancer.

## 2. Structure of PIM3 Kinase

**Figure 1:**
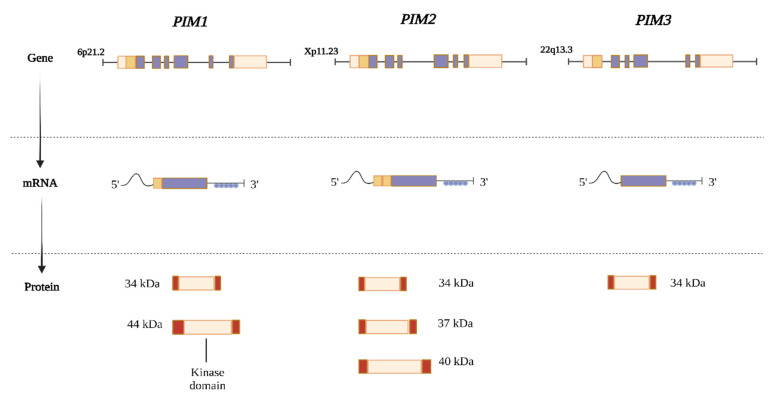
The PIM family of genes is located in several chromosomal regions in humans. Exons are indicated by purple boxes, a G/C-rich region (dark orange boxes), and five copies of the AUUUA destabilizing motif in their extensive 5′ and 3′ untranslated regions (UTRs) are depicted as blue circles. Alternative translation initiation sites and extra codons found at the 5′ of these mRNAs are used to generate various protein isoforms (dark orange boxes). Despite having various molecular weights, all PIM protein isoforms still exert serine/threonine kinase activity. PIM family kinases also share a great deal of similarity. PIM1 and PIM3 are 71% identical, and PIM3 shares 44% amino acid sequence homology with PIM1 and PIM2 [23].

Kinases are the largest human gene family and make up ~2% of the genome. PIM kinases consist of three structurally similar isoform serine/threonine kinases, including *PIM1*, *PIM2*, and *PIM3*, which are located at chromosomes 6p21.2, Xp11.23, and 22q13.3, respectively, in humans [1,7]. PIM kinases have evolutionarily conserved sequences with high homology between them [21], and the PIM3 protein shows high (71%) amino acid sequence similarity with human PIM1 and PIM2 (44%) [22]. The mRNA transcripts for *PIM* are encoded by six exons with large 5′ and 3′ untranslated regions, containing a G/C-rich region and five copies of AUUA-destabilizing motifs, respectively [7]. This results in mRNA transcripts that encode various PIM protein isoforms, all of which retain their serine/threonine kinase activities [23] (Figure 1).

In contrast to most kinases, PIM kinases are regulated mainly at the transcriptional and translational levels rather than by membrane recruitment or phosphorylation [23]. PIM kinases are also regulated by proteasomal degradation since they lack a regulatory domain and remain in active conformation when expressed [1,24]. The PIM1 kinase gene encodes two isoforms, 34 kDa and 44 kDa, which have similar kinase activities in vitro and are regulated by alternative initiation sites [25]. Alternative initiation sites for PIM2 have also been reported, resulting in the production of three distinct isoform proteins at 34, 37, and 40 kDa, whereas the *PIM3* transcript only codes for a single protein (34 kDa) [1].

Independent groups have reported crystal structures for PIM1 and PIM2, but the crystal structure of PIM3 remains undetermined [21,26]. Members of the PIM family exhibit substrate specificity and vary in their tissue distribution despite sharing similar amino acid compositions. Different malignancies exhibit isoform-specific patterns of PIM kinase overexpression. PIM2 is expressed in myeloma, lymphoma, and leukemia, whereas PIM1 is detected in many types of solid tumors and blood cancers. PIM3, on the other hand, is usually observed in adenocarcinomas of the pancreas, colon, and liver, as well as in melanoma, glioblastoma, and TNBC, and has been reported to play a significant role in cell proliferation, cell cycle regulation, and apoptotic signaling [7,15,16,17,18,19,20].

## 3. PIM3 Is Overexpressed in Solid Cancers

Pim-3 is overexpressed particularly in tumor tissues of endoderm-derived organs such as the liver, pancreas, and colon, as well as in nasopharyngeal carcinoma, prostate and breast cancer, and some sarcomas (i.e., Ewing’s sarcoma) [27,28]. Moreover, PIM3 protein is barely detected in normal adult endoderm-derived organs, such as the liver, pancreas, colon, and stomach, but its expression is elevated in premalignant and malignant lesions in these organs [12,13,16]. Elevated levels of PIM3 have also been observed in precancerous lesions of the liver, specifically in regenerative nodules and adenomatous hyperplasia. Compared to adenocarcinoma tissues, colon and stomach adenoma tissues have higher levels of PIM3 [13,16]. Recently, enhanced PIM3 mRNA expression was found in TNBC cells [20]. *PIM3* expression has been observed in normal tissues such as the liver, periphery of the pancreas, secretory epithelium of the stomach, and intestinal epithelium in mouse embryos through in situ hybridization. These observations suggested that PIM3 plays a significant role in the initial phases of carcinogenesis in several solid cancers. Additionally, *PIM3* has been detected in the kidneys, lungs, thymus, and central nervous system of mouse embryos [29]. *PIM* knockout mice are fertile; however, they have significantly reduced body size at birth and impaired responses to hematopoietic growth factors in vitro [30]. These studies indicate that PIM proteins are critical for the proliferation of T lymphocytes mediated by synergistic T-cell receptor and interleukin-2 signaling and indicate that members of the PIM family of proteins are important but dispensable factors for growth factor signaling.

## 4. Preclinical Studies of PIM3 in Solid Cancers

Accumulating evidence suggests that PIM3 plays critical roles in many cellular processes, including cell proliferation, survival, and tumorigenesis, in some solid tumors. *PIM-3* silencing reduces the growth of several types of cancer cells in vitro by inducing apoptosis [8,12,13] (Table 2). PIM3-transgenic mouse-derived hepatocytes showed faster cell cycle progression. In PIM3 transgenic mice, injection of diethylnitrosamine, a powerful hepatocarcinogen, resulted in rapid proliferation of liver cells in the early phase, as well as lipid droplet formation with increased proliferating cell numbers in the later phase, when compared to wild-type mice. Furthermore, PIM3 transgenic mice showed a higher incidence and burden of HCC and larger intratumoral vascular regions than wild-type mice [31]. These data suggest that PIM3 alone cannot induce HCC but can accelerate its development by modifying cell cycle progression. Another study showed that PIM3 knockdown or inhibition with AZD1208 resulted in lower cell survival, attachment-independent proliferation, and motility. In addition, AZD1208 inhibited tumor growth in a hepatoblastoma xenograft mouse model. This study showed that the inhibition of PIM kinase reduced human hepatoblastoma tumorigenicity both in vitro and in vivo [32]. Nakano et al. developed a PIM3 inhibitor (Compound **11**) using PIM1’s crystal structure as a surrogate to offer a foundation for rational drug design and showed that inhibition of PIM3 kinase activity reduced the proliferation of various pancreatic cancer cell lines. In a mouse xenograft model, compound **11** reduced the development of human pancreatic cancer tumor growth (PCI66) with minimal body weight loss [33]. PIM3 was constitutively expressed in SW480 sarcoma cells, and its inhibition by short hairpin RNA induced apoptosis. PIM3 knockdown inhibited Ser (112) but not Ser (136) phosphorylation of pro-apoptotic Bad molecules. Furthermore, in human colon cancer tissues, PIM3 co-localized with Bad in all cases and with phospho-Ser (112) Bad in the majority of cases. These findings demonstrate that PIM3 can inactivate Bad by phosphorylating it on Ser (112) in human colon cancer cells, thereby preventing apoptosis and promoting cancer growth [13]. In a study using gastric cancer cells, bufothionine inhibited growth, damaged cell membranes, and induced apoptotic cell death. PIM3 knockdown also greatly increased the anti-growth and pro-apoptotic effects of bufothionine in gastric cancer cells. In contrast, ectopic PIM3 expression significantly reduced the anti-neoplastic activity of bufothionine. Bufothionine therapy also reduced the expression of PIM3 in xenograft tumor tissues.

PIM3 was found to be expressed in a variety of human Ewing’s family tumor cell lines and forced PIM3 expression using a retroviral vector, increasing anchorage-independent growth. Moreover, co-expression of a kinase-deficient PIM3 mutant inhibited EWS/FLI1-mediated NIH 3T3 carcinogenesis in immunodeficient mice [28]. In a study using lung cancer cell line A549, Fan et al. showed that in the PIM3-deficient group, STAT3 phosphorylation, cyclin D1, and Bcl-2 levels decreased, whereas p21 and Bax levels increased. Cell proliferation was considerably suppressed (*p* < 0.05), with an increase in the fraction of G0/G1-phase cells, a decrease in S-phase cells, and a large increase in early apoptotic cells [34]. These results suggest that PIM3 downregulation is directly related to the active status of the lung STAT3 signaling system, inhibiting cell growth and inducing apoptosis.

Liu et al. studied the effect of PIM3 on the migration and invasion of melanoma. They silenced PIM3 using a short hairpin RNA (sh-PIM3) in a B16F10 melanoma cell line and discovered that sh-PIM3 decreased B16F10 cell migration and invasion in vitro. In a tumor-bearing mouse model, sh-PIM3 dramatically reduced lung metastasis of B16F10 melanoma cells. Sh-PIM3 reduced metastasis by modulating gene expression associated with EMT. PIM3 promotes the phosphorylation of STAT3, which stimulates the production of Slug, Snail, and ZEB1, thereby enhancing EMT-related alterations and inducing melanoma migration and invasion [35].

Chang et al. identified a compound (M-110) as a PIM kinase family inhibitor (predominantly PIM3). Both the prostate cancer cell line DU-145 and the pancreatic cancer cell line MiaPaCa2 express activated STAT3 (Tyr705). Treatment of DU-145 cells with M-110 or SGI-1776, a structurally unrelated PIM inhibitor, drastically reduced pSTAT3 (Tyr705) expression while leaving STAT3 expression unchanged. They employed PIM3-specific siRNA and discovered that in DU-145 cells, knocking down PIM3 but not PIM1 or PIM2 resulted in considerable downregulation of pSTAT3 (Tyr705). M-110 and SGI-1776 had no effect on STAT5 phosphorylation of Tyr694 in 22Rv1 cells, implying that pSTAT3 (Tyr705) is the target [36]. These findings demonstrated that PIM3 inhibitors decreased cancer cell proliferation by decreasing the expression of pSTAT3 (Tyr705). Wang et al. demonstrated that PIM3 increased the proliferation of human pancreatic cancer cells in vitro and in vivo and found that PIM3 is essential for the in vivo vasculogenesis of primary human pancreatic cancers using retroviral vectors PIM3 and a kinase-dead mutant PIM3 (K69M)-infected human pancreatic cancer cell line MiaPaCa-2 and an orthotopic mouse model of pancreatic cancer [37]. Another study on pancreatic cancer investigated the role of PIM3 in vivo and the underlying PIM3 signaling regulatory mechanisms using established MiaPaca-2 cells overexpressing wild-type PIM3 or K69M-PIM3 cells. A previous study revealed that cells stably overexpressing wild-type PIM3 had functionally enhanced Bad phosphorylation at Ser112 and increased proliferation. In contrast, stable inactivation of PIM3 with K69M-PIM3 or suppression of PIM3 expression with PIM3 shRNA resulted in functionally reduced phosphorylation of Bad at Ser112 and an increase in the number of apoptotic cells. After subcutaneous injection of these stable cell lines, nude mice treated with PIM3-overexpressing cells produced 100% subcutaneous tumors. However, in mice injected with PIM3 kinase inactive cells, intratumoral neovascularization and tumor cell proliferation were reduced. Furthermore, PIM3 overexpression increased intratumoral levels of p-STAT3 (Try705), p-survivin (Thr34), HGF, EGF, FGF-2, and VEGF [38]. Quan et al. established a mouse xenograft model by injecting PIM3-depleted glioblastoma cells into nude mice to study tumor formation in vivo and discovered that PIM3 is substantially expressed in human glioblastoma cell lines. Furthermore, PIM3 knockdown with shRNA resulted in decreased proliferation, cell cycle arrest in the G0/G1 phase, and enhanced apoptosis in glioblastomas. PIM3 knockdown effectively suppressed the development of glioblastoma cells transplanted subcutaneously in vivo. They also discovered that PIM3 knockdown decreased the levels of the anti-apoptotic protein Bcl-xl and cell cycle regulatory proteins such as cyclin D1 and Cdc25C while increasing the levels of the pro-apoptotic protein Bax [39]. In a study conducted with a TNBC cell line and xenograft model, targeted PIM3 by siRNA suppressed cell proliferation, migration, and invasion while inducing chemosensitivity and apoptotic cell death. PIM3 overexpression in MDA-MB-231 cells promoted proliferation, migration, and invasion. In vivo targeting of PIM3 using siRNA-nano formulations suppressed the growth of MDA-MB-231 and MDA-MB-436 tumors in immune-deficient mice [20].

**Table 2:** In vitro and in vivo studies regarding PIM3 in various types of malignancies.

Tumor Type	Methods of PIM Inhibition	Experimental Model	Effects	Ref.
Hepatocellular carcinoma (HCC)	PIM3 shRNA	In vitro	Reduces the in vitro growth of different hepatocellular carcinoma cell types by causing apoptosis	[8]
	Human PIM3 transgene	In vitro and in vivo	PIM3 transgenic mice had a higher incidence (80%) and a more severe burden of HCC. Hepatocytes show faster cell cycle progression and increased phosphorylation of Bad112	[31]
Hepatoblastoma	PIM3 siRNA and pan-PIM inhibitor (AZD1208)	In vitro and in vivo	Cell survival, attachment-independent growth, and motility were all reduced when PIM3 was inhibited by siRNA or the pan-PIM inhibitor AZD1208	[32]
	PIM3-specific inhibitor (compound 11)	In vitro and In vivo	Cell survival, attachment-independent growth, and motility were all reduced when PIM3 was inhibited by siRNA or PIM inhibitor	[33]
Colon carcinoma	PIM3 shRNA	In vitro	Reduced phosphorylation Ser112 on Bad in human colon cancer cells, preventing apoptosis and contributing to cell survival and cell proliferation	[13]
Gastric cancer	Bufothionine and PIM3 knockdown (RNAi)	In vitro and in vivo	Bufothionine’s anti-proliferative and pro-apoptotic actions in GC cells were markedly enhanced by PIM3 knockdown	[40]
Ewing’s sarcoma	Retroviral vector- overexpression of human PIM3 and a kinase-dead mutant of human PIM3 (K69M)	In vitro and in vivo	PIM3 expression induced anchorage-independent growth. In mice lacking an immune system, co-expression of a kinase-deficient PIM3 mutant reduced the growth of the NIH 3T3 tumor	[28]
Lung adenocarcinoma	PIM3 siRNA	In vitro	PIM3 downregulation induced apoptosis and inhibited cell growth; it was related to the lung STAT3 signaling pathway’s level of activity	[34]
Melanoma	PIM3 shRNA	In vitro and in vivo	PIM3-induced STAT3 phosphorylation leads to Slug, Snail, and ZEB1 expression, which exacerbated EMT-related alterations and accelerated cell migration and invasion	[35]
Pancreatic cancer	Retroviral vectors for human PIM3 overexpression and a kinase-dead mutant of human PIM3 (K69M)	In vitro and In vivo	PIM3 can stimulate the VEGF pathway, which can lead to tumor angiogenesis and proliferation	[37]
	Retroviral vectors, PIM3 shRNA, or scrambled shRNA	In vitro and in vivo	PIM3 kinase induced tumor neovascularization and tumor growth, as well as in accelerating growth of human pancreatic cancer	[38]
	PIM3 inhibitors (M-110)	In vitro	Reduced expression of pSTAT3^Tyr705^ and suppressed cancer cell proliferation	[36]
Glioblastoma	PIM3 shRNA and PIM3-depleted glioblastoma cells	In vitro and in vivo	Increased apoptosis, cell cycle arrest, and lower proliferation In vitro. In vivo, suppressed growth of glioblastoma cells inserted beneath the skin, lowering the levels of Bcl-XL, cyclin D1, and Cdc25C and raising Bax protein levels	[39]
TNBC	siRNA and lentivirus vector-based human PIM3 overexpression in TNBC cells	In vitro and in vivo	Targeting PIM3 by siRNA- inhibited cell proliferation, migration, and invasion while inducing chemo sensitivity and apoptotic cell death. PIM3 overexpression promoted proliferation, migration, and invasion.In vivo targeting PIM3 using siRNA-nano formulations suppressed tumor growth of MDA-MB-231 and MDA-MB-436 tumors in immune-deficient mice	[20]

## 5. Signaling Pathways Induced by PIM3

*PIM* genes are activated by mitogenic stimuli and are induced in response to a variety of growth factors and cytokines, which activate the JAK/STAT pathway [41], leading to the phosphorylation of the cytoplasmic receptor domain by JAK kinase and the recruitment of STATs and other signaling proteins [1].

**Figure 2:**
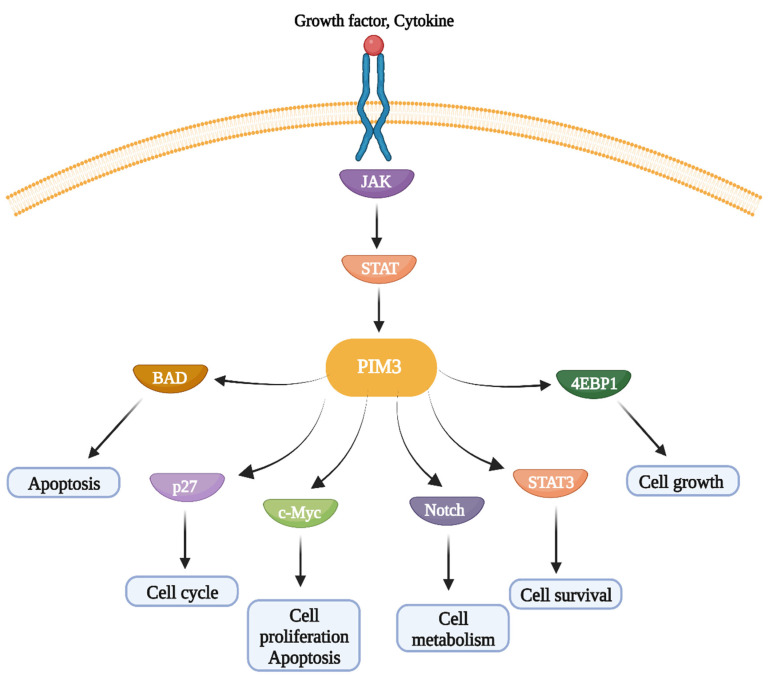
Downstream signaling pathways regulated PIM3 kinase. PIM3 regulates apoptosis, cell cycle, cell proliferation, cell metabolism, cell survival, and cell growth through activation of BAD, p27, c-Myc, Notch, STAT3, and 4EBP1 pathways activation.

PIM kinases contribute to tumorigenesis through the activation of several downstream signaling pathways (Figure 2), including phosphorylation of BAD, regulation of cell cycle progression by downregulating p27 expression and NOTCH, induction of protein synthesis by phosphorylating 4EBP1, and regulation of the oncogenic transcription factor c-Myc through its transcriptional activity [42]. The BAD protein displays pro-apoptotic activity when heterodimerized with anti-apoptotic proteins such as Bcl-2 and Bcl-XL. PIM3-induced phosphorylation releases Bcl-2 and Bcl-XL, leading to inhibition of apoptosis [7]. Recently, several reports have shown that PIM3 promotes cell invasion and migration by phosphorylating STAT3 [35]. Overall, owing to its contribution to oncogenic signaling, PIM3 is a viable molecular target that suppresses cancer cell proliferation, survival, invasion, tumor growth, metastasis, and progression.

### 5.1. STAT3

STAT3 is a transcription factor that is activated through tyrosine phosphorylation on residue 705, leading to its dimerization and translocation into the nucleus, where it binds to the DNA regulatory region of target genes [43]. STAT3 regulates cell survival, proliferation, differentiation, EMT, and apoptosis by controlling Bcl-XL, Bcl-2, Mcl-1, CyclinD1, and c-Myc [44]. The activation of STAT3 also increases the expression of Slug, Snail, and ZEB1, resulting in EMT-related changes that promote cell invasion and metastasis. PIM3 directly binds to the transcription factor STAT3 and enhances its phosphorylation [35]. To explore how PIM3 controls cell proliferation, the cell cycle, and apoptosis in lung adenocarcinoma at the molecular level, Fan et al. determined that reducing the activity of PIM3 greatly inhibits phosphorylation of STAT3, alters the distribution of cell cycle phases, and triggers apoptosis [34]. PIM3 silencing has also been observed to reduce STAT3 phosphorylation without affecting STAT3 expression in a liver cell line [45]. Additionally, in prostate cancer (DU45) and pancreatic cancer (MiaPaCa-2) cell lines, PIM3 specifically, but not PIM1 and PIM2, regulates p-STAT3 levels, potentially through its protein tyrosine phosphatase and tyrosine kinase activity [36]. These findings suggest that PIM3 regulates STAT3 and influences the expression of apoptosis-related genes (Bcl-Xl, CyclinD1, and c-Myc), as well as VEGF. In turn, this can affect proliferation, differentiation, angiogenesis, and apoptosis, contributing to the tumorigenesis of some solid cancers.

### 5.2. c-Myc

c-Myc is a proto-oncogenic transcription factor involved in cell proliferation, differentiation, and apoptosis and is often upregulated in about 70% of human cancers. PIM kinases phosphorylate serine (Ser62) and threonine (Thr58) residues of the c-Myc protein and regulate its activity [46]. Phosphorylation of Ser62 stabilizes c-Myc and leads to increased transcription and oncogenic activity, whereas reduced phosphorylation leads to c-Myc ubiquitination and degradation. Moreover, PIM3 can increase the expression of c-Myc mRNA by activating PGC-1α [47]. Thus, PIM kinases can induce tumor growth by regulating c-Myc activity [48,49,50]. Inhibition of PIM kinases has been observed to induce caspase-independent cell death in c-Myc-induced lymphomas by reducing c-Myc phosphorylation at Ser62 [51,52,53]. PIM3 is overexpressed in Burkitt’s lymphoma cell lines and lymphomas in *c-Myc* transgenic animals. As a result, PIM3 increases c-Myc levels and activity and promotes carcinogenesis [48,49]. Tumor cells with constitutively elevated Myc escape from apoptosis in the context of loss of control mechanisms (e.g., p14ARF or p53 mutation, MDM2 overexpression), along with the gain of pro-survival signals (e.g., Bcl-2 and NF-κB activation). Thus, the regulation of c-Myc activity by PIM kinases plays a critical role in tumor growth and progression. Direct inhibition of c-Myc has been shown to trigger rapid tumor regression in mice with fully reversible side effects, suggesting that regulation of c-Myc by directly targeting it or through inhibition of PIM kinases is a viable targeted therapeutic strategy in cancer.

### 5.3. BAD

Bcl-2-associated death promoter (BAD) is a pro-apoptotic protein that primarily regulates apoptosis by regulating the activity of anti-apoptotic proteins such as Bcl-XL and Bcl-2 [54]. BAD proteins heterodimerize with Bcl-2 and Bcl-XL. PIM3-induced phosphorylation of BAD releases Bcl-2 and Bcl-XL, leading to the inhibition of apoptosis. When BAD is unphosphorylated, it attaches to Bcl-2 and Bcl-XL and deactivates their anti-apoptotic function. However, several survival signals can phosphorylate BAD at Ser112, Ser136, and Ser155 residues, leading to the inactivation of BAD’s ability to induce apoptosis [55]. Owing to their homology, PIM1, PIM2, and PIM3 can increase Bcl-2 activity and promote cell survival by phosphorylating BAD at Ser112 [56]. PIM3 has been shown to be the primary factor driving colon and pancreatic cancer growth by inhibiting the pro-apoptotic activity of BAD by inducing phosphorylation at BAD Ser155 [6].

### 5.4. p27

The CDK inhibitor p27(Kip1) has a tumor suppressor function and plays an important role in regulating cell proliferation, differentiation, and apoptosis. PIM3 binds to p27 and phosphorylates threonine-157 and threonine-198 residues, leading to p27 binding to the 14-3-3 protein, which facilitates its nuclear export and proteasome-dependent destruction. PIM3 also inhibits p27 expression at the transcriptional and post-translational stages to promote cell cycle progression, proliferation, and tumor growth. The co-expression of PIM3 (+), c-Myc (+), and p-p27 (+) was closely correlated with poor differentiation, advanced tumor stage, and lymph node metastasis [57]. p27 suppresses the cell cycle at the G1 phase in conjunction with the CDK2/cyclin complex, whereas reduced p27 expression leads to cell cycle progression [58]. Studies suggest that apoptosis is induced during p27-induced G1 or S cell cycle arrest and accelerates apoptosis. Additionally, PIM kinase controls the activity of transcription factors like FOXO1a (Forkhead Box O1 a) and FOXO3a (Forkhead Box O3 a), which, upon phosphorylation, renders them inactive and suppresses the transcription of p27 [59].

### 5.5. 4EBP1

PIM kinases are involved in cell growth by controlling protein translation via regulation of the activity of 4EBP1 and p70S6K. Studies have suggested that PIM kinases enhance the activity of mTORC1, a serine/threonine kinase that regulates cellular growth and metabolism. Protein synthesis is induced by the mTORC1 complex, which consists of mTOR, Raptor, GL, and PRAS40. Phosphorylation of 4EBP1 at Ser 65 is required for the activation of the translational initiation complex. Phosphorylation of 4EBP1 at Ser 65 prevents 4EBP1 from binding to eIF4E and, hence, promotes cap-dependent translation. In addition, the pro-apoptotic activity of 4EBP1 is reduced by S65 phosphorylation [60]. A group of small-molecule inhibitors, including benzylidene-thiazolidine-2-4-diones, has been shown to inhibit PIM kinase activity in vitro at very low concentrations, reduce 4EBP1 phosphorylation, inhibit cell growth, and induce cell death in cancer cells [47]. The study found that reduced phosphorylation of 4EBP1, specifically p70S6K and its substrate S6, was consistently accompanied by growth suppression in the examined cell lines and patient samples following PIM inhibition [61].

### 5.6. NOTCH 1/3

The Notch protein family is composed of four isoforms, Notch1, Notch2, Notch3, and Notch4, which play roles in epithelial-to-mesenchymal transition and stem cell maintenance [62]. The Notch pathway contributes to chemotherapy resistance and survival in several cancers, including prostate and hematological malignancies [63]. PIM kinases phosphorylate and increase Notch1 activity, which, in turn, can increase PIM expression. Additionally, c-Myc, p21, and NF-κB are downstream targets shared by PIM kinases and Notch. In pancreatic cancer cell models, the inhibition of PIM alone (using DHPCC-9) or Notch (using DAPT) reduced tumor volumes. Co-targeting of both molecules was more successful than monotherapy, potentially benefiting patients with PIM and/or Notch pathway abnormalities [64]. Notch1 expression not only leads to epithelial-to-mesenchymal transition but also increases glycolytic metabolism. In breast and prostate cancer cells, all PIM kinases can phosphorylate Notch1 at Ser-2152, resulting in localization of the Notch1 intracellular domain in the nucleus and an increase in the transcription of Notch1 target genes [65]. PIM-mediated phosphorylation of Notch1 in breast cancer cells regulates cell metabolism, whereas Notch inhibition enforces glycolytic metabolism by impairing mitochondrial function [66]. This conclusion is supported by the observation that PIM kinases can regulate the expression of PGC-1 and c-Myc, leading to the maintenance of mitochondrial integrity and the regulation of glycolysis and mitochondrial biogenesis [47]. Prostate cancer cells, which depend less energetically on glycolysis, do not exhibit comparable metabolic effects [67]. According to Santia et al., the inhibition of PIM by phosphorylating Notch1 effectively slowed the growth of tumors, and inhibiting PIM and Notch together showed greater antitumor effects [65].

Overall, given that the activity of both PIM kinases and Notch1 plays a crucial role in driving tumor growth and progression, attempts are being made to target or co-target these molecules for targeted cancer therapy [1,68,69], which may result in new and potent cancer treatment strategies.

### 5.7. Other Targets

The inhibition of STAT3 signaling has been linked to the induction of apoptosis and suppression of survivin protein production in breast cancer cells [70]. According to a previous study, deactivating PIM3 kinase or preventing PIM3 protein production did not affect the overall survivin expression in human pancreatic cancer cells. However, it also reduced the levels of phosphorylated STAT3 at Try705. The phosphorylation of survivin at Thr34 acts as a barrier to prevent cells from undergoing apoptotic cell death [71]. Liu et al. demonstrated that PIM3 kinase inactivation or reduction of PIM3 protein expression lowered survivin’s Thr34 phosphorylation levels, while PIM3 overexpression boosted them, indicating that PIM3 regulates survivin activity to promote cell survival [38]. Expression of the kinase-dead PIM3 mutant reduced CD31-positive areas, whereas overexpression increased vascularity in MiaPaca-2 pancreatic cancer tumor xenografts [37]. Later, Liu et al. showed that PIM3 overexpression enhanced VEGF expression [38]. Furthermore, PIM3-overexpressing cells considerably enhanced EGF and FGF expression compared to parental cells, but PIM3 kinase activity loss markedly decreased EGF and FGF mRNA expression. Consequently, neovascularization/angiogenesis and subsequent tumor growth may be attributed to the signals transmitted by EGF and FGF. Tumors expressing an inactive PIM3 kinase exhibit reduced HGF mRNA expression [38]. The lack of phenotypic changes caused by hereditary PIM3 loss raises the possibility that PIM3 is physiologically unnecessary. Unlike other survival kinases, such as Akt kinases, PIM kinases are not downstream of the insulin receptor signaling pathway, so their inhibition has little effect on the pathway. When developing anticancer drugs to target solid tumor angiogenesis, it is beneficial to focus on inhibiting the production of PIM3, as it is often overproduced in these situations. Inhibition of PIM3 can promote tumor cell death and prevent angiogenesis, leading to a dual effect on tumor growth [38].

## 6. Therapies Targeting PIM in Cancer

Because of the oncogenic nature of PIM kinases, there has been increasing interest in developing small-molecule inhibitors that directly target the adenosine triphosphate (ATP)-binding domain of PIM proteins for cancer treatment [72]. Currently, there is no specific inhibitor of PIM3; however, several inhibitors have been developed as pan-PIM inhibitors that inhibit PIM1, PIM2, and PIM3, including SGI-1776, AZD1208, and PIM447, and these inhibitors have been used in clinical trials (Table 3). Clinical trials underway for PIM kinase inhibitors are shown in Table 4.

### 6.1. SGI-1776

SGI-1776 is a non-specific small-molecule inhibitor that inhibits all three PIM kinases (PIM1–3) in addition to FLT3 (a cytokine receptor) and Haspin (a serine/threonine kinase). The IC_50_ values were found to be as 7nM, 363nM, and 69nM for PIM1, -2, and -3, respectively, in chronic lymphocytic leukemia cells [73]. SGI-1776 inhibited FLT3 and Haspin at nanomolar concentrations. SGI-1776 was found to be an effective cytotoxic drug in several publications for a variety of cancers, including CLL [73], prostate cancer [74], renal cell carcinoma [75], AML [76], and multiple myeloma [77]. Additionally, it has been demonstrated that SGI-1776 can be used in combination with other drugs to inhibit PIM kinase proteins, which increases the cytotoxicity of sunitinib and cytarabine against renal cell carcinoma and AML, respectively [75,78]. Cervantes-Gomez et al. also evaluated the effect of SGI-1776 on myeloma cell lines and CD138(+) myeloma cells and revealed that SGI-1776 treatment led to apoptosis in both replicating primary cells and proliferating cell lines [79]. According to Chen et al., SGI-1776 inhibits acute myeloid leukemia by reducing the transcriptional activity of Mcl-1, a member of the anti-apoptotic Bcl-2 family [73]. SGI-1776 has undergone two clinical trials, both of which were funded by Astex Pharmaceuticals (NCT01239108 and NCT00848601). Due to a dose-limiting hazard in ventricular electrical cycle (QTc) lengthening in patients with relapsed/refractory leukemia, the first was discontinued prior to enrolment, and the second inhibitor, which was effective against all three PIM kinases, was discontinued for treating patients with prostate cancer and lymphoma because of its cardiac toxicity. This indicates that it is not a safe or clinically useful option for patients [63].

### 6.2. AZD1208

AstraZeneca developed AZD1208, an orally administered pan-PIM kinase inhibitor. It has low nanomolar activity against all three PIM isoforms in vitro and potent activity in cells [80]. AZD1208 demonstrated antitumor effects by targeting multiple pathways, including apoptosis, proliferation, translation, and vesicular transport. AZD1208 has been investigated in preclinical models of prostate cancer [81], acute myelogenous leukemia (AML) cell lines, and xenograft tumor models [82]. In AML cell lines, AZD1208 inhibited the growth of 5 of 14 AML cell lines. This sensitivity was correlated with the expression of PIM1 and STAT5 activation. AZD1208 caused cell cycle arrest and apoptosis in MOLM-16 cells by decreasing the phosphorylation of BAD, 4E-BP1, p70S6K, and S6 proteins while increasing cleaved caspase 3 and p27 [82]. Studies have suggested that the mTOR pathway plays a role in the AZD1208 mechanism of action, explaining how AZD1208 suppresses tumor growth in mice with MOLM-16 and KG-1a. Furthermore, the use of AZD1208 decreased colony formation and the presence of PIM-targeted proteins in primary AML cells in the bone marrow, regardless of whether they had the FLT3 wild type or FLT3 internal tandem duplication mutant. Additionally, treatment with AZD1208 led to a reduction in the proliferation of non-Hodgkin lymphoma and prostate cancer cells through the inhibition of PIM kinase and c-Myc-mediated activity [81,83]. Clinical trials in patients with AML, malignant lymphoma, and solid malignancies (NCT01489722 and NCT01588548) have assessed the pharmacokinetics, effectiveness, safety, and tolerability of AZD1208. AZD1208 is well tolerated by patients with heavily treated AML and advanced solid malignancies. In two dose-escalation studies (up to 700 mg), the mean half-life after a single dose was approximately 37.2 h [84]. After multiple doses, AZD1208 increased CYP3A4 activity, leading to an increase in drug clearance. The effectiveness of using AZD1208 alone to fight tumors is not fully understood because it greatly increases CYP3A4 activity and does not show much response in clinical settings. Consequently, the development of AZD1208 was terminated. However, using AZD1208 to inhibit PIM kinase could be a useful anticancer strategy when combined with other treatments.

### 6.3. PIM447 (LGH447)

Novartis has developed PIM447, a small-molecule inhibitor that targets the PIM kinase family of enzymes. This inhibitor demonstrated significant kinase selectivity across all PIM isoforms in kinome screening studies, with Ki values of 0.006 nM for PIM1, 0.018 nM for PIM2, and 0.009 nM for PIM3 [85]. PIM447 is currently being evaluated in clinical trials as a potential treatment for various types of cancers. In a clinical trial aimed at treating relapsed/refractory multiple myeloma, PIM447 was found to be well tolerated. The trial showed a disease control rate of 72.2%, a clinical benefit rate of 25.3%, and an overall response rate of 8.9%. Unfortunately, the trial had to be terminated because of disease progression in 54 of the 79 patients who participated. Bone marrow examination revealed no significant reduction in the number of malignant plasma cells. These findings suggest that PIM447 exerts a cytostatic effect [86]. Previous research has suggested that PIM inhibitors may be more effective in combination with other therapies. They have shown limited success as monotherapy for both hematological and solid tumors. Currently, PIM447 is being tested in combination with ruxolitinib, a JAK1/2 inhibitor, and LEE011, a selective CDK4/6 inhibitor, in patients with myelofibrosis (NCT02370706). However, this study was terminated early after 15 patients were recruited into the dose-escalation study owing to unfavorable hematologic toxicity. A phase 1/2 study (NCT02144038) aimed to investigate the safety and effectiveness of combination therapy with PIM447 and BYL719 (PI3K-alpha inhibitor) in patients with relapsed and refractory multiple myeloma. The study was terminated because the treatment was not well tolerated by the patients.

Another clinical study (NCT02078609), which combined PIM447 with midostaurin, was also terminated because of minimal antitumor activity. The examination of pharmacokinetic (PK) results also revealed a complex drug–drug interaction between PIM447 and midostaurin, preventing consistent and predictable concentrations of both drugs. Despite the termination of the study, no safety concerns prompted the decision. The maximum tolerated dose for PIM447 was not determined, but doses of 250 mg or 300 mg once daily appeared to be safe and well tolerated in Japanese patients with multiple myeloma. The pharmacokinetic exposure of PIM447 increased with dose, and there was no apparent impact on CYP3A4 activity. Single-agent antitumor responses were observed when PIM447 was administered at a dose of 250 mg or 300 mg once daily, as demonstrated by an overall response rate of 15.4%, a clinical benefit rate of 23.1%, and a disease control rate of 69.2%. Overall, PIM447 clinical trials are in the early stages, and more research is needed to determine the safety and efficacy of PIM447 in treating cancer. However, the results from preclinical studies and initial clinical trials are promising and suggest that PIM447 may have potential as a treatment for various types of cancers.

## 7. PIM3 and Immunotherapy

**Figure 3:**
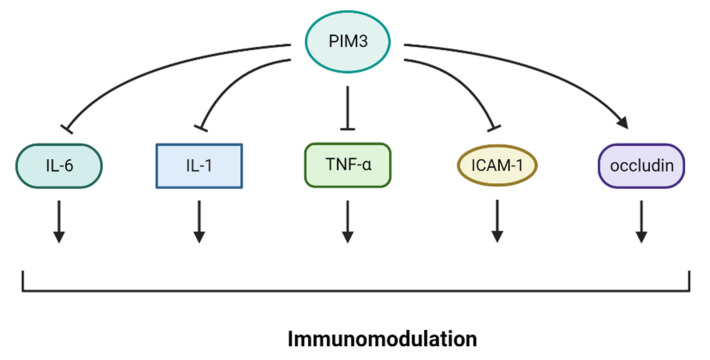
PIM3 regulates immunomodulators. PIM3 inhibits the expression of IL-6, IL-1, TNF-α, and ICAM-1 and encourages the expression of occluding.

Recent studies have suggested a potential connection between PIM3 kinase and response to immunotherapy and the role of PIM kinases in inducing immunosuppression in hematological and solid cancers. PIM 1–3 proteins are overexpressed in B-cell malignancies, such as mantle cell lymphoma [87], diffuse large B-cell lymphoma (DLBCL), Burkitt’s lymphoma [88], chromosome 6-acquired non-Hodgkin’s lymphoma [89], and chronic lymphocytic leukemia [90]. PIM kinase family proteins are associated with poor prognosis in patients with DLBCL. In B-cell malignancies, PIM kinase expression is associated with c-Myc [91] and nuclear factor-kappa B (NF-ĸB) [92], as well as CD40 and PDL1 expression, both of which induce immunosuppression [93], suggesting that targeting PIM kinases may enhance the efficacy of immunotherapies [94]. PIM kinase inhibitors have been studied in combination with immunotherapy. In fact, these findings suggest that combining PIM kinase inhibitors with PD1 inhibition improves T-cell response to immunotherapy. PIM inhibition, when combined with an anti-PD1 antibody, results in long-lasting tumor control in addition to increasing the central memory phenotype of T cells. Overall, PIM3 inhibition may enhance the clinical efficacy of immunotherapy [95]. Pan-PIM kinase (PIM1 + PIM3) inhibition in T cells is linked to the expression of memory-related genes that are inversely correlated with glycolysis and glucose uptake. Moreover, pan-PIM kinase inhibition decreased the expression of CD38, a negative regulator of T-cell metabolic fitness. Importantly, blocking PIM kinases in tumor-bearing animals receiving adoptive T-cell therapy and enhancing this combination with anti-PD1 antibody significantly increases the efficacy of anticancer T-cell treatment [95]. PIM3 inhibited the expression of IL-6, IL-1, TNF-α, and ICAM-1 and promoted the expression of occludin (Figure 3), reducing pancreatic acinar cell damage by LPS. PIM3 may preserve damaged pancreatic acinar cells by controlling inflammatory reactions in the surrounding tissues [96].

Analysis of the correlation between the immune signatures of pancreatic cancer and PIM1, PIM2, and PIM3 revealed that PIM expression was significantly correlated with higher MAPK activation scores, T-cell inflammation scores, inflammatory markers, MHC class I and II gene expression, and various immune cell infiltrations. Thus, a comprehensive understanding of these variations can lead to customized treatment options for pancreatic cancers expressing PIM [97]. It is important to note that the connection between PIM3 kinase and immunotherapy may be critical for enhancing the efficacy of immunotherapy, and further studies are needed to fully understand the precise mechanisms and therapeutic implications and to provide valuable insights into the potential benefits and limitations of targeting PIM3 kinase in the context of immunotherapy.

## 8. PIM3 and Resistance to Chemotherapy and Radiotherapy

PIM kinases have been found to induce resistance to a variety of anticancer treatments, including chemotherapy and radiotherapy. PIM1 overexpression is associated with a poor response to radiotherapy [98,99], and PIM3 overexpression induces resistance to platinum- and taxane-based chemotherapies [10]. PIM may control drug transporters, which may contribute to its ability to resist chemotherapy-induced apoptosis. Treatment with PIM inhibitors sensitizes cells to chemotherapy by regulating ATP-binding cassette (ABC) drug transporters and reduces chemotherapy-induced apoptosis [100]. Mounting evidence suggests that cancer cells routinely modify their biology and evade apoptosis using PIM kinases as a survival mechanism. The role of PIM3 in the chemoresistance of PDAC was shown through either genetic or pharmacological inhibition (i.e., SGI-1776), suggesting that PIM3 inhibition could be a potential strategy to sensitize PDAC cells to gemcitabine and improve treatment outcomes in patients with this aggressive cancer [101].

Furthermore, targeting PIM3 kinase and its role in signaling pathways is being investigated as a potential therapeutic strategy to overcome drug resistance in cancer treatment. PIM1 and PIM3 have been shown to confer resistance to PI3K inhibitors [102] and different types of inhibitors, such as PI3K/mTOR dual inhibitors, PDH1 inhibitors, Akt inhibitors, and several mTOR inhibitors. PIM expression seems to be related to the mechanism of acquired resistance in MET inhibitor-resistant clones as the pan-PIM kinase inhibitor AZD1208 inhibits the development of resistant colonies despite having no effect on the growth of cancer cells, suggesting that PIM kinases play a role in the development of resistance to MET inhibitors [103].

In the context of radiotherapy, ionizing radiation has been found to induce PIM3 in pancreatic cancer cells, leading to radioresistance in both in vitro and in vivo tumor models [104]. Stable overexpression of PIM3 in pancreatic cancer cells dramatically reduced the DNA damage response and G2/M-phase cell cycle arrest, protecting cells from radiation therapy. Silencing PIM3 expression led to an increase in H2AX phosphorylation, a DNA double-strand break marker, and a decrease in ataxia-telangiectasia-mutated kinase activation. Thus, radiation may not be as effective against cancers overexpressing PIM3. Overall, combining radiation with PIM3 targeting agents may increase the effectiveness of radiotherapy in patients with pancreatic cancer. More detailed studies are needed to uncover the exact mechanisms by which PIM3 may confer radiotherapy resistance [104]. Overall, developing PIM3-specific inhibitors or combination therapies that target PIM3 along with other resistance-related pathways could be a promising approach to improving cancer treatment outcomes.

## 9. Conclusions and Future Prospects

PIM3 proto-oncogene is frequently overexpressed in various human cancers, including hematological malignancies and solid tumors, and its expression is associated with poor clinical outcomes and shorter patient survival. Recent studies indicate that PIM3 plays a pivotal role in cancer proliferation, survival, invasion, metastasis, angiogenesis, tumor growth and progression, chemo/radiation resistance, and the immunosuppressive microenvironment. Targeting PIM3 delays tumor growth in in vivo tumor models with no side effects, suggesting that PIM3 is an excellent therapeutic strategy for some solid cancers. Despite ongoing clinical trials exploring PIM kinase inhibitors, there are currently no FDA-approved pan-PIM kinase or specific PIM3 inhibitors. Although several pan-PIM inhibitors have been tested in clinical trials, they are not selective enough to eliminate adverse effects. Further research is required to develop highly selective, potent, and safe PIM3 inhibitors. In conclusion, based on recent studies on various solid cancers, PIM3 has emerged as a promising therapeutic target. The identification of safe, selective, potent, and effective PIM3 inhibitors provides a foundation for clinical translation and may have a significant clinical impact on the treatment of some solid and hematological cancers by offering new hope for patients with PIM3-driven cancers.

## Figures and Tables

**Table 1:** List of human cancers and association of increased expression of PIM3 levels with clinical outcome.

Cancer Types	Association of High PIM3 Levels with Clinical Outcome			Ref.
	Poor survival	Metastases	Tumor size/stage	
Ovarian cancer	−	+	+	[15]
Gastric cancer	+	−	−	[16]
Colorectal carcinoma	+	+	−	[17]
Prostate cancer	+	+	+	[5]
Pancreatic cancer	+	+	+	[18]
Hepatocellular carcinoma	+			[19]
Triple-negative breast cancer	+	+	+	[20]

PIM3 levels were higher in malignant tissues relative to healthy tissues in each of the tumor types. PIM3 overexpression was correlated with poor patient survival, metastases, and/or tumor size/stage (+). However, correlation was not found or determined in some cases (−).

**Table 3:** The status of clinical trials for PIM kinase inhibitors and their activity. IC50: Half maximal inhibitory concentration, Ki: inhibitory constant.

Inhibitors	IC50/Ki (nM)		
	PIM1	PIM2	PIM3
SGI-1776	7	363	69
AZD1208	0.4	5	1.9
PIM447	0.006	0.018	0.009

**Table 4:** Summary of Clinical trials for PIM kinase inhibitors.

Drug	Cancer Type	Clinical Trial Phase	Clinical Trial Registry Number	Results
SGI-1776 (Astex Pharmaceuticals)	Relapsed/Refractory Leukemia	Phase 1 (withdrawn)	NCT01239108	Withdrawn due to cardiac QTc prolongation
	Refractory Prostate and Relapsed/Refractory Non-Hodgkin’s Lymphoma	Phase 1 (terminated)	NCT00848601	Withdrawn due to cardiac QTc prolongation
AZD1208 (Astrazeneca)	Acute Myelogenous Leukemia (AML) Patients	Phase 1 (terminated)	NCT01489722	Serious AEs in 23 patients (discontinued in 8) and DLTs in 5; no clinical response
	Advanced Solid Tumors and Malignant Lymphoma	Phase1 (completed)	NCT01588548	Serious AEs in 8 patients and DLTs in 4; best response SD ≥ 6 weeks (13 patients; ORR, 0%)
PIM447 (Novartis)	Relapsed and Refractory Multiple Myeloma	Phase 1/2 (completed)	NCT02144038	A maximum tolerated dose was not declared, and the phase II portion of the study was not initiated
	AML or High-Risk Myelodysplastic Syndrome (MDS)	Phase 1 (completed)	NCT02078609	Data revealed minimal antitumor activity, (PK) results also revealed a complex drug–drug interaction between PIM447 and midostaurin
	Multiple Myeloma	Phase 1 (completed)	NCT02160951	MTD for expansion was not determined, an overall response rate of 15.4%, a clinical benefit rate of 23.1%, and a disease control rate of 69.2%
	Myelofibrosis	Phase 1 (terminated)	NCT02370706	Terminated due to hematologic toxicity
	Relapsed and/or Refractory Multiple Myeloma	Phase 1 (completed)	NCT01456689	Hematologic grade 3/4 AEs; disease control rate of 72.2%, ORR of 8.9%

Abbreviations: AE, adverse event; DLT, dose-limiting toxicity; MDS, myelodysplastic syndrome; MTD, maximum tolerated dose; QTc, heart rate-corrected QT interval ORR, overall response rate.
